# Permutation tests for hypothesis testing with animal social network data: Problems and potential solutions

**DOI:** 10.1111/2041-210X.13741

**Published:** 2021-10-28

**Authors:** Damien R. Farine, Gerald G. Carter

**Affiliations:** ^1^ Department of Evolutionary Biology and Environmental Studies University of Zurich Zurich Switzerland; ^2^ Department of Collective Behavior Max Planck Institute of Animal Behavior Konstanz Germany; ^3^ Centre for the Advanced Study of Animal Behaviour University of Konstanz Konstanz Germany; ^4^ Department of Ecology, Evolution, and Organismal Biology The Ohio State University Columbus OH USA; ^5^ Smithsonian Tropical Research Institute Balboa, Ançon Panama

**Keywords:** animal social networks, hypothesis testing, permutation tests, social behaviour, social network analysis

## Abstract

1. Permutation tests are widely used to test null hypotheses with animal social network data, but suffer from high rates of type I and II error when the permutations do not properly simulate the intended null hypothesis.
2. Two common types of permutations each have limitations. Pre‐network (or datastream) permutations can be used to control ‘nuisance effects’ like spatial, temporal or sampling biases, but only when the null hypothesis assumes random social structure. Node (or node‐label) permutation tests can test null hypotheses that include nonrandom social structure, but only when nuisance effects do not shape the observed network.
3. We demonstrate one possible solution addressing these limitations: using pre‐network permutations to adjust the values for each node or edge before conducting a node permutation test. We conduct a range of simulations to estimate error rates caused by confounding effects of social or non‐social structure in the raw data.
4. Regressions on simulated datasets suggest that this ‘double permutation’ approach is less likely to produce elevated error rates relative to using only node permutations, pre‐network permutations or node permutations with simple covariates, which all exhibit elevated type I errors under at least one set of simulated conditions. For example, in scenarios where type I error rates from pre‐network permutation tests exceed 30%, the error rates from double permutation remain at 5%.
5. The double permutation procedure provides one potential solution to issues arising from elevated type I and type II error rates when testing null hypotheses with social network data. We also discuss alternative approaches that can provide robust inference, including fitting mixed effects models, restricted node permutations, testing multiple null hypotheses and splitting large datasets to generate replicated networks. Finally, we highlight ways that uncertainty can be explicitly considered and carried through the analysis.

## 1 INTRODUCTION

Permutation tests are arguably among the most useful statistical tools for the modern biologist. They are commonly used in ecology (Gotelli & Graves, 1996), biogeography (Harvey, 1987), community ecology (Miller et al., 2017) and in studies of ecological networks (Dormann et al., 2009) and social networks (Croft et al., 2011). Permutation tests randomise (or re‐assign) observed data to generate a distribution of statistic values expected under a given null hypothesis. Researchers create case‐specific null models by permuting data in specific ways (e.g. constraining permutations within specific groups) while keeping other aspects of the dataset the same (e.g. where and when observations were made). They are particularly useful when the standard assumptions of other statistical tests are violated, as is often the case with social network data (see Farine, 2017 for a general introduction). However, several recent studies (Evans et al., 2020; Puga‐Gonzalez et al., 2021; Weiss et al., 2021) have highlighted issues with using social network data to test null hypotheses about the relationship between a predictor and a response (i.e. conducting regressions using network data).

The first issue is caused by the fact that different kinds of permutations create different null hypotheses. *Pre‐network permutations* (or datastream permutations) are used to test how observed social network structure differs from what is expected if animals made random social decisions. This approach permutes the observed data to create many expected networks that could have occurred in the absence of any social preferences. The null hypothesis here is that, after removing the specified effects, the social structure itself is random (e.g. individuals have no other social preferences). This is a different null hypothesis than what researchers typically want when performing a correlation or regression with network data (Weiss et al., 2021). To test the statistical significance of a correlation or regression, a common permutation approach is to use *node permutations* (or node‐label permutations), as used in Mantel tests and the quadratic assignment procedure (QAP) tests. By only permuting the node labels, this approach removes the statistical relationship, while preserving the same observed network properties in all expected networks. For a typical node permutation test, the null hypothesis is that there is no statistical relationship between a predictor (e.g. kinship) and response (e.g. association rate) in the *observed* network, which is correct for regression‐based questions.

Drawing inferences from the observed network is, however, a challenge for animal social data. In the social sciences, the observed social network often accurately reflects affiliations, relationships or rates of contact. In many ecological studies of animal behaviour, by contrast, the *observed* social network is typically a non‐random sample that does not directly or even accurately represent the ‘real’ social network of dyadic social preferences or contact (Farine & Whitehead, 2015). Constructing animal social networks requires large numbers of observations (Farine & Strandburg‐Peshkin, 2015; Davis et al., 2018; Langen, 1996) with many repeated observations of the same individuals to accurately infer social relationships. Further, the observed network is typically shaped simultaneously by multiple confounding ‘nuisance effects’—that is, biological and methodological factors besides the hypothesised effect of interest. For example, the structure of an observed social network could be shaped primarily by individual site preferences, habitat constraints on movement or methodological biases. An individual animal (node) may be less connected to others in the observed network only because it is harder to observe or identify, it uses a smaller subset of sampled locations, it has its main home range outside the study area, or it left the study population early. Another example is that individuals at the edge of a study area (compared to individuals at the centre) might have many associations with individuals that were never observable. For further discussion, see *Illustrating the Drivers of Type I and II Error* in Appendix S1.

Sampling biases will vary in magnitude and importance across study designs (Davis et al., 2018), but they are often inevitable. Even automated methods such as proximity sensors (Ripperger et al., 2020; Ryder et al., 2012) or barcodes (Alarcón‐Nieto et al., 2018; Crall et al., 2015) are not free of sampling biases if animal‐borne proximity sensors vary in their sensitivity (e.g. due to tiny differences in soldering) or if some barcodes are more difficult to identify by computer vision. Of course, unreliable observations, sampling biases and other nuisance effects are not unique to animal social data, but these problems are especially troubling for social network analyses because observations are not independent (e.g. a single under‐sampled node affects all the edges with all other nodes). The interacting roles of biological and methodological nuisance effects on the observed network structure can be difficult to identify and disentangle, and failing to do so can easily lead to spurious inferences (Farine & Aplin, 2019).

Nuisance effects can be accounted for using a range of approaches. They could be estimated using covariates or random effects in a parametric model within a frequentist or Bayesian frameworks, or it is possible to control for nuisance effects using null models, for example by constraining permutations within blocks of time or space. There are many potential advantages to the parametric approach including an ability to explicitly measure the magnitude of each effect and rely less on *p*‐values (Franks et al., 2021; Hart et al., 2021). However, as most existing statistical models of social network data have been developed in the social sciences, where observed social networks are relatively unbiased representations of the real network, it remains unclear how well—or easily—these approaches can cope with the multiple kinds of nuisance effects common in observational studies of animal behaviour. Capturing all nuisance effects in the model becomes increasingly challenging as the number of interacting nuisance effects increases, and when they do not have regular patterns across time or space. For example, one cannot simply fit individuals' locations as a random effect by assigning individuals to a singular spatial location when home ranges are continuously distributed and overlapping in space. Furthermore, once the network has been created, the nuisance effects generally cannot be recovered from the network itself.

The second common approach involves using pre‐network permutations to simultaneously control for multiple potential nuisance effects by constraining swaps of the data to occur within blocks of time or space (Farine, 2017; Spiegel et al., 2016; Sundaresan et al., 2009; Whitehead, 2008; Whitehead et al., 2005). In doing so, the null model can hold constant many features of the observed data including group sizes, number of observations per individual, individual variation in space use (and therefore spatial overlaps with all others), temporal autocorrelation in behaviour, temporal overlap among all pairs of individuals, the distribution of demographic classes across space and time, the variation in the density of individuals across space and time, and differences in sampling effort across space and time. Problems arise, however, when testing the relationships between a predictor and response because pre‐network permutations simulate a completely different null hypothesis (i.e. random social structure outside the specified effects). As a result, the permuted networks do not have socially realistic network‐level properties of real animal societies, such as the natural variance in edge weights or distribution of degree values. Therefore, when a social network has other sources of social structure beyond the effect of interest, pre‐network permutation tests will produce highly elevated false positives when testing the significance of a correlation or regression (Puga‐Gonzalez et al., 2021; Weiss et al., 2021).

**FIGURE 1:**
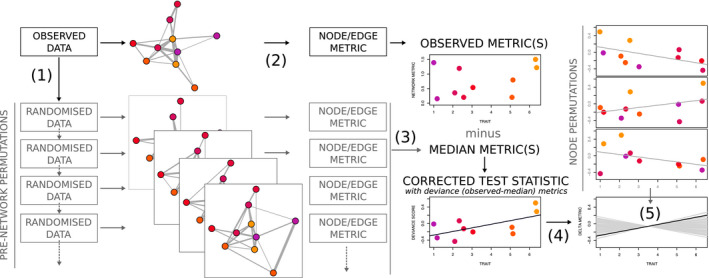
Overview of the double permutation method. First, pre‐network permutations (1) generate a null distribution of expected metric values for a unit of interest (e.g. a node's degree or an edge's weight) alongside each unit's observed metric (2). Next, to remove nuisance effects for each unit, the median of expected metric values is subtracted from the observed metric value, yielding a corrected metric value called the ‘deviation score’ (3). Then, to generate a corrected test statistic, we fit a model with the deviation scores (4). To generate a *p*‐value, a nonparametric node permutation test can then be used to compare the corrected and expected test statistics (5). Alternatively, an appropriate parametric model could be used to replace (4) and (5)

Our goal in this paper is to address this limitation of pre‐network permutation tests, allowing them to be applied to regression while robustly accounting for multiple common nuisance effects. We propose an initial solution, which we show can maintain rates of both false positives and false negatives to around 5% under a range of scenarios. Our approach (Figure 1) uses pre‐network permutations to account for nuisance effects by constraining swaps of observations within blocks of time and space, and then node permutations to conduct a nonparametric test for the effects of *X* on *Y*. This ‘double permutation’ approach tests the null hypothesis that there is no relationship between a predictor and deviations from random social structure (within the specified temporal and spatial constraints). Specifically, we use pre‐network permutations to estimate the deviation of each unit's observed measure from its expected random value (e.g. the difference between a node's observed degree and the median values of the same node's degree across the permuted networks). We call this observed–expected difference the deviation score (e.g. ∆degree). These deviation scores, which account for nuisance effects, are then fit into a model of interest to generate a test statistic, and a node permutation test provides the *p*‐value for the test statistic.

Using deviation scores is conceptually similar to generalised affiliation indices (Whitehead & James, 2015), but, rather than fitting covariates in a regression model, the deviance of each unit's metric from random expectations are extracted from pre‐network permutations. Similar to extracting residuals from a model, this approach subtracts a measure of central tendency of the randomised data from the observed data, because no single permuted network can be considered as the true expected random network. As a measure of central tendency for the expected values, we use the median because the distributions of expected values are often highly non‐normal and long tailed. However, use of the median assumes that the expected values have a unimodal distribution. To check this assumption and other properties such as symmetry, we recommend visualising the distribution of expected values generated by the pre‐network permutation (see *Potential Improvements* in Appendix S1).

We call this a ‘double permutation’ because we enter the deviation scores extracted from the pre‐network permutations into a node‐label permutation test (e.g. QAP), but one could also opt to enter the deviation scores into an appropriate parametric model. Indeed, the basic procedure can be applied to any model for calculating test statistics, such as those generated from regressions, Mantel tests (Mantel, 1967), network regression models like multiple regression quadratic assignment procedure (MRQAP; Dekker et al., 2007) and metrics such as the assortativity coefficient (Farine, 2014). We also show that the double permutation approach performs well with ‘gambit‐of‐the‐group’ association data and with data collected using focal observations. We acknowledge that this is only one potential and imperfect solution, and we therefore also highlight alternative hypothesis‐testing methods that are worth evaluating further.

## 2 TESTING THE ROBUSTNESS OF THE DOUBLE PERMUTATION APPROACH

We evaluate the performance of the double permutation tests when using gambit‐of‐the‐group data (simulation 1), focal sampling data (simulation 2) and dyadic observations (simulation 3), both in the absence of any real relationship and when there are strong nuisance effects. In simulations 1 and 2, we simulate the process of testing for a relationship between an individual trait and a social network metric, using three common weighted metrics (see Farine & Whitehead, 2015): weighted degree (or strength), eigenvector centrality and betweenness. In simulation 3, we simulate the process of testing for a link between pairwise kinship and association rate in a species that exhibits other social preferences not based on kinship. For each simulated dataset, we calculate *p*‐values using several tests: node permutations, node permutations in which the model controls for covariates (number of observations and, where possible, location), pre‐network permutations, pre‐network permutations on the t‐statistic (the equivalent of scaling the predictions, as proposed by Weiss et al., 2021), pre‐network permutations in which the model controls for covariates (number of observations and, where possible, location) and the double permutation method. We create the networks and conduct the permutation tests using the r package asnipe (Farine, 2013) and use the package sna (Butts, 2008) to calculate network metrics.

### 2.1 Simulation 1: Regression between a node metric and a trait using gambit of the group data

We first simulate a researcher using group‐based observations to test whether individuals with a given trait value are more gregarious under three biological scenarios: (a) individuals choose groups at random, (b) individual choice is predicted by a social trait (*T*_S_), whereby individuals with higher trait values choose to be in larger groups and (c) individual choice is predicted by a spatial preference (*L*_S_), whereby individuals with higher trait values are not more social but prefer sites with more resources that also hold more individuals. The last scenario represents an alternative driver of gregariousness that is challenging to control in model fitting or using node permutations by location because individual spatial preferences cannot be reduced down to a single value. Such variation in spatial distribution of individuals is common, for example the number of great tits *Parus major* in Wytham Woods varied 10‐fold across different parts of the woodland (Farine et al., 2015). In each scenario, we simulated 100 replications for varying combinations of network size (5–120 individuals) and mean numbers of observations per individual (5–40). For each simulation (one network), we extract *β* coefficients (slopes) and *t* statistics by fitting the model, weighted metric ~ trait (+ covariates where applicable), using the lm function in r. Note that pre‐network permutations inherently control for the number of observations of each individual, and we also control for where observations were made by restricting swaps to within locations. Further details for simulation 1 are in Appendix S1.

### 2.2 Simulation 2: Estimating differences in group means using focal sampling data

We next simulate a researcher using focal sampling data to test whether different classes of individuals vary in their gregariousness. We build on the code of Puga‐Gonzalez et al. (2021) and Farine (2017), whereby each simulation assigns individuals to observations, with each observation having a focal individual and connections made between the focal and each individual observed. Simulations can be run with and without a sex difference in gregariousness. When the difference is present, females are made more gregarious by being disproportionately present in observations containing many individuals. The simulations can also introduce an observation bias, whereby females are often not observed even when present, whereas males are always observed when present. Such biases are common in field studies—for example in a study on vulturine guineafowl *Acryllium vulturinum* (Papageorgiou et al., 2019), juveniles are marked on the right wing and are only identifiable if their right side is observed, whereas adults are marked with leg bands that can be identified from any direction. The code runs 500 simulations for each scenario (sex effect or not, observation bias present or not), with parameter values that are randomly drawn from uniform distributions as follows: population size ranging from 10 to 100, observation bias ranging from 0.5 to 1 (where 1 is always observed), the female sex ratio ranging from 0.2 to 0.8 and the number of focal follows ranging from 100 to 2,000. The simulation procedure was designed to allow an estimate of false positive rates (when no effect should be present but one is detected) and false negative rates (when an effect is present, but masked by the observation bias, and therefore not detected), and of the effect sizes before and after the observation bias occurs. For each simulation (one network), we extract *β* coefficients and *t* statistics by fitting the model, weighted metric ~ sex (+ covariates), in the lm function in r. Simulation 2 has been used in several studies to estimate performance of permutation approaches (Farine, 2017; Puga‐Gonzalez et al., 2021) as it can capture both the pre‐ and post‐biased metrics.

### 2.3 Simulation 3: Regression between edge weight and a dyadic relationship such as kinship using dyadic observations

Finally, we simulate a researcher testing for a link between kinship and social network structure, where edges are association rates that are driven by both kinship and other social preferences. To evaluate the impact of other social preferences on error rates, we generate simulated networks in which individuals have three types of social associates: (a) weak associates, (b) preferred associates and (c) strongly bonded associates. Our simulations comprise two scenarios. In the first, association rates (edge weights) are independent of kinship, which we achieve by randomising the kinship matrix after generating it. In the second, associations are kin biased such that strongly bonded associates have higher kinship on average and weak associates and non‐associates (missing edges) have on average lower kinship. For each type of social network and scenario, we created ‘real’ social networks and then create ‘observed’ social networks by simulating observations. We simulate 100 replications for combinations of network sizes, with 5–120 individuals and the mean numbers of observations per individual ranging from 5 to 40. For each simulation (one network), we conduct a MRQAP with the model: edge weight ~ kinship (+ covariates). Further details for simulation 3 are in Appendix S1.

## 3 THE DOUBLE PERMUTATION APPROACH IS ROBUST TO TYPE I AND TYPE II ERRORS

Our simulations confirm that the double permutation method is robust to type I errors across a range of scenarios. In simulation 1 (Table 1), pre‐network permutation tests suffer from elevated false positives (type I error rate of 26%), consistent with previous studies (Evans et al., 2020; Puga‐Gonzalez et al., 2021; Weiss et al., 2021). We further show that the tendency for pre‐network permutation tests to generate type I errors is greater in smaller networks and when more data are collected (Figure S2, see also Evans et al., 2020). Pre‐network permutations using the *t* value as a test statistic are also prone to false positives, and perform relatively poorly at detecting a true effect (detecting them consistently less often than other approaches). Combining pre‐network permutation tests together with model structures that also control for nuisance effects, when estimating the test statistic, also appears to produce unreliable results. As expected, node permutation tests perform particularly poorly when the effects are driven by non‐social nuisance effects, such as variation in the spatial distribution of individuals, even when controlling for individuals' locations in the model. The parametric *p*‐values produced similar results to the node permutations. When node permutations were applied to a more informed model, the *p*‐values were not clearly better or worse.

**TABLE 1:** Propensity for different permutation tests to yield errors or detect real effects when using regression models to test hypotheses on networks collected using gambit‐of‐the‐group data (model 1). Table shows the proportion of statistically significant results for an effect of a trait on degree under three sets of scenarios. When no effects are present, the expected proportion of significant results should be 5%. When a social effect is present, most results should be significant. When a spatial confound is present, the proportion of significant results should again approach 5%. Controlled node permutations include the number of observations in the models plus individuals' most common location in the spatial confound condition. Figures S2–S4 show how the proportion of significant results is affected by the number of observations and the number of nodes in the network

	No effects (should be low)	Social effect only (should be high)	Spatial confound (should be low)
Degree			
Node permutation (*β*)	4.8%	88.4%	88.4%
Controlled node permutation (*β*)	4.8%	90.4%	47.0%
Pre‐network permutation (*β*)	26.0%	90.2%	23.9%
Pre‐network permutation (*t*)	13.7%	65.3%	29.3%
Controlled pre‐network permutation	26.0%	89.7%	23.9%
Double permutation	4.7%	89.7%	10.8%
Eigenvector centrality			
Node permutation (*β*)	5.1%	93.2%	92.5%
Controlled node permutation (*β*)	5.1%	93.6%	63.6%
Pre‐network permutation (*β*)	34.1%	95.3%	39.7%
Pre‐network permutation (*t*)	15.2%	76.6%	22.3%
Controlled pre‐network permutation	34.3%	95.0%	23.1%
Double permutation	4.9%	93.4%	33.6%
Betweenness			
Node permutation (*β*)	4.6%	86.3%	86.5%
Controlled node permutation (*β*)	4.9%	85.6%	72.9%
Pre‐network permutation (*β*)	20.2%	74.5%	69.3%
Pre‐network permutation (*t*)	11.2%	72.5%	69.7%
Controlled pre‐network permutation	19.9%	74.3%	65.6%
Double permutation	4.9%	70.9%	67.0%

The double permutation test performs generally better than the other methods we tested across the three scenarios, producing conservative *p*‐values when no effect is present, detecting the relationship between a trait and social metrics when present, and being more conservative than other tests when the effect is driven by non‐social factors.

All tests appeared to struggle with confounded measures of eigenvector centrality and betweenness, but this is likely driven by the simulations not always producing datasets in which eigenvector centrality was strongly linked to spatial location, thereby largely over‐estimating the false positive rate. However, because all methods were tested on identical simulated datasets, their relative performance can still be meaningfully compared.

In simulation 2 (Table 2), pre‐network permutations again show elevated type I error rates in the absence of a true difference between classes of individuals and node permutations again show elevated type I error rates in the presence of nuisance effects. Controlling for nuisance effects in the model with node permutations helps under some circumstances (when there is a strong effect and an observation bias), but not others (e.g. when there is no effect and a bias, or when combined with pre‐network permutation tests). The double permutation test almost always performs as expected. One exception is the high levels of type II errors for betweenness. This may occur because betweenness is an unstable metric (i.e. adding one edge can substantially change the distribution of betweenness values in the whole network) and pre‐network permutations are therefore not generating a meaningful null distribution on which the node permutation can operate.

**TABLE 2:** Propensity for permutation tests to produce type I and type II errors from datasets simulating focal sampling (model 2). Simulations comprise four scenarios: (a) females and males have identical social phenotypes and are observed equally, (b) females are more social and both sexes are observed equally, (c) females and males have identical social phenotypes but observations are biased towards males (20% of observations of females are missed), and (d) females are more social but observations are biased towards males (20% of observations of females are missed)

	No observation bias		Observation bias (‘nuisance’ effect)	
	Phenotypes equal (Type I errors)	Females more social (Type II errors)	Phenotypes equal (Type I errors)	Females more social (Type II errors)
Degree				
Node permutation (*β*)	5.0%	1.2%	60.4%	37.8%
Controlled node permutation (*β*)	5.6%	20.0%	68.2%	10.4%
Pre‐network permutation (*β*)	37.8%	5.2%	34.2%	7.4%
Pre‐network permutation (*t*)	30.8%	69.0%	60.0%	36.2%
Controlled pre‐network permutation	39.0%	24.2%	69.6%	12.4%
Double permutation	5.2%	6.6%	7.0%	6.0%
Eigenvector centrality				
Node permutation (*β*)	4.8%	2.4%	55.0%	31.6%
Controlled node permutation (*β*)	7.0%	2.4%	71.4%	7.0%
Pre‐network permutation (*β*)	45.2%	1.2%	43.0%	3.0%
Pre‐network permutation (*t*)	23.4%	74.4%	64.4%	34.6%
Controlled pre‐network permutation	38.8%	14.0%	79.2%	5.2%
Double permutation	4.8%	4.2%	6.2%	7.0%
Betweenness				
Node permutation (*β*)	5.8%	8.6%	67.0%	52.8%
Controlled node permutation (*β*)	4.6%	34.4%	33.6%	93.2%
Pre‐network permutation (*β*)	17.0%	69.2%	19.0%	84.6%
Pre‐network permutation (*t*)	15.9%	68.7%	18.3%	79.9%
Controlled pre‐network permutation	17.2%	69.4%	19.8%	82.0%
Double permutation	4.6%	86.2%	5.2%	26.2%

Simulation 3 (Table 3) shows that the double permutation test can reliably test null hypotheses that assume nonrandom social structure (similar to a node permutation test like QAP), such as whether association rates are predicted by kinship in cases where kinship effects are present or absent. As with the above two simulations, the double permutation test performs well when no real effect is present (i.e. type I error rates were close to 5%). All models in this simulation have elevated type II error rates because not all simulated networks with the added effect actually resulted in data with a clear effect, but the double permutation test performs more conservatively than node permutations (producing more type II errors).

**TABLE 3:** Propensity for permutation tests to produce type I and type II errors regarding kinship effects from simulated datasets with confounding social effects, that is, nonrandom social structure (model 3). Table shows the type I error rates in simulations where the social effect is a confound (i.e. strong associations are not linked to kinship), and estimated type II error rates in simulations where the social effect corresponds to the hypothesis being tested (i.e. strong associations are linked to kinship). Figures S5 and S6 show how the proportion of significant results is affected by the number of observations and the number of nodes in the network. While pre‐network permutations appear to outperform other approaches with respect to Type II errors, this is likely because they are also more sensitive to weak effects in small networks, which are likely to correspond to type I errors rather than correctly identifying a true effect (see Figure S6)

	Kinship ≠ Associations (Type I errors)	Kinship ∝ Associations (Type II errors)
Node permutation (*β*)	5.1%	12.5%
Controlled node permutation (*β*)	4.9%	12.0%
Pre‐network permutation (*β*)	18.6%	6.2%
Pre‐network permutation (*t*)	3.0%	79.1%
Double permutation	5.2%	16.5%

## 4 RECOMMENDATIONS

Our results confirm that pre‐network permutations by themselves cannot be used to generate a *p*‐value for a correlation, regression or any comparison of means measured from the observed network. Having an understanding of the system and data collection procedure, and considering the possible nuisance effects that might create differences between the observed and actual network, is always critical. If the observed network is indeed an accurate reflection of the real network, then node permutations, the double permutations test described here, or any well‐specified parametric models can be used (how to do this is beyond the scope of this paper).

Our results suggest that across scenarios, the double permutation test often performs better at testing null hypotheses using regressions than the other approaches when there are strong confounds, such as sampling biases or spatial preference drivers. These create high rates of false positives in tests that do not include pre‐network permutations. However, the double permutation test can be too conservative when applied to unstable global node metrics such as betweenness (Table 2), which might be better studied using node permutations (in the absence of confounding effects). Because the suitability of permutation methods has not been exhaustively tested with measures of betweenness, we cannot recommend any solution for hypothesis testing with betweenness in the presence of nuisance effects. Our recommendations for choice of permutation test across node metrics and scenarios are summarised in Table 4.

**TABLE 4:** Recommendations from simulations for choice of permutation tests. In the absence of nuisance effects, or when weak nuisance effects can effectively be controlled in a node permutation (e.g. using restricted node permutations), then either node permutations or double permutations are likely to provide robust inference for most local network metrics (e.g. degree, eigenvector centrality) or for relational data (tests on edge weights). In the presence of nuisance effects, double permutations tests are generally recommended, except for betweenness

	Metric			Relational (edge weight)
	Degree centrality	Eigenvector centrality	Betweenness centrality	
No clear nuisance effects	Node or double	Node or double	Node	Node or double
Nuisance effects expected	Double	Double	(Unclear)	Double

Despite our focus on permutation tests, one should not infer from this study that permutation tests (or other nonparametric tests) are necessarily superior to parametric models. Carefully fitting a parametric model (or a generative model) that can explicitly capture all effects (both effects of interest and nuisance effects), identified through careful inspections of residuals and other model diagnostics, can have many benefits (see alternative approaches). For better or worse, one benefit of nonparametric tests is that they avoid the process of having to specify the best possible statistical model to fit the data. A permutation test simply compares observed and expected model coefficients (in this case extracted from the lm and MRQAP functions in r) as descriptive metrics rather than as interpretable parameters of biological meaning. However, this limits inference to null hypothesis testing, rather than estimation of the magnitude and confidence of effect sizes (Franks et al., 2021).

## 5 THE CHALLENGE OF CALCULATING EFFECT SIZES WITH SAMPLING BIAS

Inference will always benefit from relying less on *p*‐values and instead focusing more on estimating effect sizes (Nakagawa & Cuthill, 2007), and the same is true for network data (Webber et al., 2020). Permutation tests do not, in and of themselves, provide interpretable effect sizes, meaning that the interpretation of the biology arising from the test is limited to the *p*‐value. Franks et al. (2021) show that, under certain conditions, the coefficients of regression models applied to network data can generate reliable relative effect sizes after controlling for the number of observations. Multiple confounded nuisance effects can create more difficult problems when using models to estimate effect sizes. We explore this using simulation 2, where one category of individuals is both more social and less observable. Such a situation is common in nature, for example female birds that are more drab than males (e.g. most sexually dimorphic birds), shyer birds that are more social and more difficult to detect (Aplin et al., 2013), subadults that are more central but more difficult to recognise than adults (e.g. because they have fewer markings or as in the guineafowl example cited above). Gregariousness and detectability can be linked if individuals vary in their habitat preferences; for example, cooperatively breeding striated thornbills *Acanthiza lineata* are more social than the brightly coloured scarlet robins *Petroica boodang* that they flock with, but also more arboreal and camouflaged resulting in fewer observations per individual (Farine & Milburn, 2013).

In simulation 2, the original coefficient (before the observation bias) and estimated coefficient (with the observation bias) are correlated (*r* = 0.54), yet controlling for the number of observations of each individual consistently inflates the estimated coefficient size (Figure S7). We then test whether regression models can recover the original coefficient value using two approaches to fitting the number of observations as a covariate. First, we use a naïve model, where the scaled number of observations is simply added as a covariate. Second, we use a more informed model where the number of observations is also added as an interaction with the effect of sex (since exploration of the data would show that the number of observations differs between the sexes). As expected, the naïve model performs worse, producing estimated effect sizes that are on average 1.8 times the original value (and up to 5.1 times the original value), but correctly fitting the interaction term does not dramatically improve the estimate, with the average estimated coefficient values being 1.7 times the original value (and up to 3.3 times the original). These two models also perform very poorly at estimating effect sizes when the true effect is not present (the estimated effect sizes were on average over 250 times the true values, Figure S8). Less reliable effect sizes will also lead to less reliable *p*‐value and hence inferences.

Nuisance effects do not need to be accounted for both within the regression model and again within the null model. Indeed, simulations show that controlling twice for nuisance effects using both covariates and permutations yields less reliable results (see results for Controlled pre‐network permutation in Tables 1 and 2). In summary, a more complex model could yield correct effect sizes, but correctly accounting for multiple nuisance effects within the model is non‐trivial. We encourage future work exploring this topic.

One reason why simple regression models struggle to generate robust effect sizes when controlling for nuisance effects might be because they do not deal well with individuals that are observed in groups, rather than in pairs, which is common in animal social network studies (Sah et al., 2019), and known to cause problems for analysis (Evans et al., 2020). For such gambit‐of‐the‐group observations, the loss of each observation can result in a variable number of edges being removed within groups. For example, when using the simple association index to estimate proportion of time spent together (Hoppitt & Farine, 2018), an individual A in a group of 10 that is imperfectly observed will have a reduction in degree of 0.9 for every 10% of decrease in its detection rate, while other perfectly detected members of the group will all decrease in degree by only 0.1 per 10% decrease in the detection rate of individual A. This difference in the effects of imperfect detections on degree among members of the same group become greater as group size increases, and is not resolved by increasing sampling. Given our findings, approaches to estimating corrected effect sizes should be carefully tested before being used. Estimating effect sizes in the presence of bias is a major priority in the continued development of robust tools for animal social network analysis.

## 6 ALTERNATIVE APPROACHES

While the double permutation test performs similarly, and usually better, than the single permutation procedures across a range of scenarios, many alternative approaches or methodological refinements can improve the robustness of inferences from hypothesis testing. Here we discuss some alternative and further approaches, but also note that not all of these methods have been exhaustively tested for performance.

### 6.1 Non‐permutation approaches

While this paper is focused on addressing existing problems with nonparametric permutation tests, there are also compelling arguments for moving away from permutation tests (Franks et al., 2021; Hart et al., 2021). Hart et al. (2021) argue that, for network regression analyses, well‐specified parametric models should be used in favour of permutation tests because mixed (or hierarchical) models and Bayesian approaches can account for nonindependence in the data (e.g. by including actors and receivers as random effects), regressions tested with node permutations can violate the exchangeability of residuals, and because parametric or Bayesian approaches will reduce or remove emphasis on *p*‐values and instead increase focus on estimating effect sizes. It would therefore be helpful to have more studies testing if and how parametric models can reliably control for common types of sampling biases and other nuisance effects described in this paper. Ideally, such studies should compare the performance of different model‐fitting and permutation procedures across a range of ecologically realistic data collection scenarios, and also assess the severity of the problem of nuisance effects under various forms of data collection.

Gimenez et al. (2019) proposed to deal with sampling biases by using capture–recapture models to explicitly model heterogeneity in detections, thereby providing more accurate estimates of network metrics. Studies estimating phenotypic variance using animal models have also proposed methods to decompose multiple sources driving between‐individual variation in trait values (Thomson et al., 2018). Such multi‐matrix models have recently been applied to animal social networks as a means of identifying the relative importance of different predictors in driving differences in social network metrics (Albery et al., 2021).

### 6.2 Restricted node permutations

For many studies, it may be sufficient to use node permutations and control for nuisance effects by restricting which individuals' data are swapped when performing the randomisation. Such restricted node permutations are useful if individuals can be easily allocated to a distinct spatial location, or if there are clear categories of individuals that correspond to biases. Say, for example, that individual animals enter the study in distinct waves because of a standard dispersal time or because a study expanded at some point to include new individuals. In this case, permutations could be restricted to only allow swaps among individuals that entered the study at approximately the same time. However, if multiple nuisance effects have to be accounted for, one can rapidly run out of sets of exchangeable individuals. For example, a study with 40 individuals that aims to restrict swaps by two parameters (e.g. age and location) would have on average only 10 individuals per class if both parameters are binary, only six to seven per class if one is trinary, and only four to five per class if both are trinary.

### 6.3 Study‐specific simulations

How can we be sure that a chosen method is effective? One approach is to explicitly test how sensitive a given dataset is to generating false positives or false negatives under different hypothesis‐testing approaches. The procedure we used here—simulating a random trait value for each node in a network and running through the full hypothesis‐testing procedure—can be a straightforward way of characterising the robustness of any given study's results. This procedure simply involves generating a random trait variable (e.g. drawing a trait value from a normal distribution) and testing how this value corresponds to the metric of interest from the observed network using the same code as for the real variable(s) being studied. By repeating this procedure many times, one can observe the proportion of the tests that incorrectly produce a significant *p*‐value. This result can be also reported as evidence of the selected method's reliability. It is worth exploring further how this study‐specific information might be used. For example, one might be able to correct the threshold for rejecting the null hypothesis to the point where the expected false positive rate will be 5%. Shuffling the actual node values and running a pre‐network permutation test (and repeating these two steps many times) might provide an even more precise estimation of the true false positive rate, as it will be fully conditioned on the real observation data.

### 6.4 Multiple null models

Testing one null hypothesis can encourage confirmation bias, and might only reject a strawman hypothesis of little interest (e.g. that chimpanzees have random interactions). Strong inference (Platt, 1964) therefore requires considering and testing multiple alternative hypotheses. Similarly, multiple null models can be used to collectively examine the different processes that might be shaping the patterns present in observation data, and can be highly informative. Multiple null models are particularly effective for generating an understanding of the effects of social decisions versus space use on social network structure (e.g. Figure S1). While it is important to control for the contribution of ‘nuisance’ spatial effects to social network structure when testing hypotheses about social decision‐making, the process by which animals use space (and its links to social structure) is itself an important biological question (He et al., 2019; Webber & Vander Wal, 2018). We show an example of this in Figure S1, where both social and spatial processes shape the differences in the social connectedness of males and females. Pre‐network permutations that control for space would discard the biological drivers of space use (and, consequently, group size) as a nuisance effect. Aplin et al. (2015) evaluated the extent that the spatial distribution of individuals contributed towards their repeatability in social network metrics by reporting the distribution of repeatability values from a spatially constrained permutation test. Farine et al. (2015) used two different permutation tests to identify the expected effects of individuals choosing social groups versus choosing habitats. Implementing multiple null models requires careful consideration of elevation in false positives (Webber et al., 2020). Looking forward, the practice of developing multiple null (or reference) models using permutation can be further extended to include generative (e.g. agent‐based) models that test specific alternative hypotheses (Hobson et al., in press).

### 6.5 Bootstrapping (and its limitations)

Another approach that is often considered to be useful for estimating uncertainty (e.g. confidence interval around effect sizes) is bootstrapping (Bonnell & Vilette, 2021; Farine & Strandburg‐Peshkin, 2015; Lusseau et al., 2008), which involves resampling the observed data with replacement to create new datasets of the same size as the original. This procedure can estimate the range of values that a given statistic can take, and whether the estimate overlaps with an expected null value (see Puth et al., 2015). Bootstrapping, however, is not always appropriate as a means of hypothesis testing in animal social networks, because like node permutations, it relies on resampling the observed data under the assumption that the observed network reflects the true social structure. For example, missing edges in an association network represent an association rate of zero, but in many cases these zero values could actually be weak associations that exist in the real world but were not observed. In such cases, bootstrapping the edge weights suggests that these unobserved edges have no uncertainty, which is obviously false. Thus, bootstrapping social network data should only be used with care.

### 6.6 Replicated networks

Pre‐network permutation tests were initially designed to evaluate whether the social structure of a population is nonrandom, given sparse association data (Bejder et al., 1998), and error rates for regression‐based hypotheses degrade as more observations are collected for a few nodes (Figure S2). The good news is that many observations allow the creation of replicated networks (Hobson et al., 2013), which is achieved by splitting the dataset to produce multiple networks (without overlapping observations). When doing so, it is important that each replicated network contains sufficient data to produce reliable estimates of network structure (Farine, 2018). The same hypothesis‐testing procedure can then be applied to each network independently. Using emerging methods for automated tracking, social networks can be created for each season (e.g. Papageorgiou et al., 2019), across periods of several days (e.g. Dakin et al., 2021), each day (e.g. Boogert et al., 2014) or even each second (e.g. Blonder & Dornhaus, 2011). Independent networks that produce consistent results when tested independently provides much stronger support for a given hypothesis than any single network can. If, instead, effects are unstable over time, this might suggest either the presence of other underlying dynamics that warrant further investigation, more careful analyses, or the need for longer time periods for each replication.

Any inference becomes stronger again if each of the replicate social networks contains different sets of individuals or if the network is reformed in each time period. An example of this are within‐roost association networks that are formed each day after foraging bats all leave and come back into the roost before sunrise (Ripperger et al., 2019). Alternatively, a hypothesis could be tested on alternative subsets of the populations, such as different communities (Bond, Konig, et al., 2021; Bond, Lee, et al., 2021). Given sufficient data, replicated networks could be combined by using tools from meta‐analyses to estimate an overall effect size. However, such an approach would need to ensure that the same biases do not impact each of the networks in the same way.

Although within‐study replication can improve our confidence in a given result, ultimately the gold standard is replication across studies, as within‐study replications cannot control for many of the nuances in how data are collected, stored and analysed. One example of a replication study tested the effects of developmental conditions on the social network position of juvenile zebra finches *Taeniopygia guttata*. In the original study (Boogert et al., 2014), birds were given either stress hormone or control treatments as nestlings, and their social relationships were studied after they became nutritionally independent from their parents. In the replication study (Brandl et al., 2019), clutch sizes of wild zebra finches were manipulated to experimentally increase or decrease sibling competition (a source of developmental stress), and social associations (in the wild) were recorded after birds fledged. Across both studies, 9 of 10 hypothesised effects had the same result (i.e. both statistically significant or not), and all 10 of the hypothesised effects were in the same direction (binomial *p* < 0.0001).

## 7 CONCLUSIONS

We propose an approach that avoids the elevated false positives that occur from pre‐network permutations assuming random network structure under the null hypothesis and node permutations assuming an unbiased observed network under the null hypothesis. Our proposed solution, or the use of permutation tests more generally, does not negate the need to carefully consider statistical issues that have been highlighted for more orthodox statistical practices (Forstmeier et al., 2017). For example, the common practice of using models for both data exploration and hypothesis testing is estimated to produce rates of type I error as high as 40% (Forstmeier & Schielzeth, 2011). High false discovery rates can also be caused by overfitting, ignoring model assumptions or choosing an incorrect model structure (e.g. by failing to fit random slopes to a mixed effects model, see Schielzeth & Forstmeier, 2009). The same pitfalls occur when using these methods to calculate a test statistic for use with a permutation test. In general, false positive rates are likely to increase with the complexity of the question and the dataset, and dealing with empirical datasets in the biological sciences often requires making complex decisions for which the solutions are not clear—such as whether to log‐transform count data and use a simple general linear model (Ives, 2015) or to fit a more complex generalised linear model (O'Hara & Kotze, 2010). In some cases, researchers may opt for a less powerful tool that may be easier to wield correctly.

In the context of null hypothesis testing with social network data, each permutation procedure (including constricting the swaps in different ways) creates a specific null model (see Figure S1 for an example), so the crucial statistical consideration is ensuring that the correct null hypothesis is being tested. One particularly important point highlighted by this work, and that of others (e.g. Franks et al., 2021), is the need to pay close attention to the importance of different processes for a given hypothesis of interest. For example, if we assume that space use is constrained by non‐social factors, and if we aim to understand animal social decisions, then space use (and its consequences) could be considered a nuisance effect. However, if we aim to study the transmission of information or pathogens, then space use is an important factor of interest contributing to the outcome of the transmission process. Thus, a given process might represent a nuisance effect for one question but not another, even if these factors represent two halves of the same feedback loop, such as habitat choices driving social preferences and vice versa (Cantor et al., 2021).

Permutation tests can control for a large range of nuisance effects within the null model without explicitly identifying, measuring and controlling for every source of bias. There are both pros and cons of this approach, but we believe that the strength and robustness of permutation tests lies in their flexibility and simplicity. By using a range of permutation tests to measure the relative deviations of the observed data from different null expectations, it is also possible to evaluate the relative contribution of different processes shaping a network. Regardless of the method used, inference with social network data requires thinking carefully about what specific processes may have produced the patterns in a given observed dataset.

## CONFLICT OF INTEREST

The authors declare no conflict of interest.

## AUTHORS' CONTRIBUTIONS

D.R.F. and G.G.C. developed the method, and wrote the manuscript together; D.R.F. developed and ran the simulations.

### PEER REVIEW

The peer review history for this article is available at https://publons.com/publon/10.1111/2041‐210X.13741.

## Supporting information

Supplementary Material

## Data Availability

This submission contains no primary data. The R function and code to replicate the simulations are here: https://doi.org/10.17617/3.7x.

## References

[mee313741-bib-0001] Alarcón‐Nieto, G. , Graving, J. M. , Klarevas‐Irby, J. A. , Maldonado‐Chaparro, A. A. , Mueller, I. , & Farine, D. R. (2018). An automated barcode tracking system for behavioural studies in birds. Methods in Ecology and Evolution, 9, 1536–1547. 10.1111/2041-210X.13005

[mee313741-bib-0002] Albery, G. F. , Morris, A. , Morris, S. , Pemberton, J. M. , Clutton‐Brock, T. H. , Nussey, D. H. , & Firth, J. A. (2021). Multiple spatial behaviours govern social network positions in a wild ungulate. Ecology Letters, 24, 676–686. 10.1111/ele.13684 33583128

[mee313741-bib-0003] Aplin, L. M. , Farine, D. R. , Morand‐Ferron, J. , Cole, E. F. , Cockburn, A. , & Sheldon, B. C. (2013). Individual personalities predict social behaviour in wild networks of great tits (*Parus major*). Ecology Letters, 16, 1365–1372. 24047530 10.1111/ele.12181

[mee313741-bib-0004] Aplin, L. M. , Firth, J. A. , Farine, D. R. , Voelkl, B. , Crates, R. A. , Culina, A. , Garroway, C. J. , Hinde, C. A. , Kidd, L. R. , Psorakis, I. , Milligan, N. D. , Radersma, R. , Verhelst, B. , & Sheldon, B. C. (2015). Consistent individual differences in the social phenotypes of wild great tits (*Parus major*). Animal Behaviour, 108, 117–127. 10.1016/j.anbehav.2015.07.016 26512142 PMC4579410

[mee313741-bib-0005] Bejder, L. , Fletcher, D. , & Brager, S. (1998). A method for testing association patterns of social animals. Animal Behaviour, 56, 719–725. 10.1006/anbe.1998.0802 9784222

[mee313741-bib-0006] Blonder, B. , & Dornhaus, A. (2011). Time‐ordered networks reveal limitations to information flow in ant colonies. PLoS ONE, 6, e20298. 10.1371/journal.pone.0020298 21625450 PMC3098866

[mee313741-bib-0007] Bond, M. L. , Konig, B. , Lee, D. E. , Ozgul, A. , & Farine, D. R. (2021). Proximity to humans affects local social structure in a giraffe metapopulation. Journal of Animal Ecology, 90(1), 212–221. 10.1111/1365-2656.13247 32515083

[mee313741-bib-0008] Bond, M. L. , Lee, D. E. , Farine, D. R. , Ozgul, A. , & Konig, B. (2021). Sociality increases survival in adult giraffes. Proceedings of the Royal Society B: Biological Sciences, 288, 20202770. 10.1098/rspb.2020.2770 PMC7893237 33563118

[mee313741-bib-0009] Bonnell, T. R. , & Vilette, C. (2021). Constructing and analysing time‐aggregated networks: The role of bootstrapping, permutation and simulation. Methods in Ecology and Evolution, 12(1), 114–126. 10.1111/2041-210X.13351

[mee313741-bib-0010] Boogert, N. J. , Farine, D. R. , & Spencer, K. A. (2014). Developmental stress predicts social network position. Biology Letters, 10, 20140561. 10.1098/rsbl.2014.0561 25354917 PMC4272205

[mee313741-bib-0011] Brandl, H. B. , Farine, D. R. , Funghi, C. , Schuett, W. , & Griffith, S. C. (2019). Early‐life social environment predicts social network position in wild zebra finches. Proceedings of the Royal Society B: Biological Sciences, 286, 20182579. 10.1098/rspb.2018.2579 PMC6408881 30963840

[mee313741-bib-0012] Butts, C. T. (2008). Social network analysis with sna. Journal of Statistical Software, 24, 1–50. 18618019 10.18637/jss.v024.i01 PMC2447931

[mee313741-bib-0013] Cantor, M. , Maldonado‐Chaparro, A. , Beck, K. , Carter, G. G. , He, P. , Hilleman, F. , Klarevas‐Irby, J. A. , Lang, S. D. J. , Ogino, M. , Papageorgiou, D. , Prox, L. , & Farine, D. R. (2021). The importance of individual‐to‐society feedbacks in animal ecology and evolution. Journal of Animal Ecology, 90, 27–44. 10.1111/1365-2656.13336 32895936

[mee313741-bib-0014] Crall, J. D. , Gravish, N. , Mountcastle, A. M. , & Combes, S. A. (2015). BEEtag: A low‐cost, image‐based tracking system for the study of animal behavior and locomotion. PLoS ONE, 10, e0136487. 10.1371/journal.pone.0136487 26332211 PMC4558030

[mee313741-bib-0015] Croft, D. P. , Madden, J. R. , Franks, D. W. , & James, R. (2011). Hypothesis testing in animal social networks. Trends in Ecology & Evolution, 26, 502–507. 10.1016/j.tree.2011.05.012 21715042

[mee313741-bib-0016] Dakin, R. , Moore, I. T. , Horton, B. M. , Vernasco, B. J. , & Ryder, T. B. (2021). Testosterone‐mediated behavior shapes the emergent properties of social networks. Journal of Animal Ecology, 90, 131–142. 32745255 10.1111/1365-2656.13305

[mee313741-bib-0017] Davis, G. H. , Crofoot, M. C. , & Farine, D. R. (2018). Estimating the robustness and uncertainty of animal social networks using different observer methods. Animal Behaviour, 141, 29–44.

[mee313741-bib-0018] Dekker, D. , Krackhardt, D. , & Snijders, T. (2007). Sensitivity of MRQAP tests to collinearity and autocorrelation conditions. Psychometrika, 72, 563–581. 10.1007/s11336-007-9016-1 20084106 PMC2798974

[mee313741-bib-0019] Dormann, C. F. , Fründ, J. , Blüthgen, N. , & Gruber, B. (2009). Indices, graphs and null models: Analyzing bipartite ecological networks. The Open Ecology Journal, 2, 7–24. 10.2174/1874213000902010007

[mee313741-bib-0020] Evans, J. C. , Fisher, D. N. , & Silk, M. J. (2020). The performance of permutations and exponential random graph models when analyzing animal networks. Behavioral Ecology, 31, 1266–1276. 10.1093/beheco/araa082

[mee313741-bib-0021] Farine, D. R. (2013). Animal social network inference and permutations for ecologists in R using asnipe. Methods in Ecology and Evolution, 4, 1187–1194.

[mee313741-bib-0022] Farine, D. R. (2014). Measuring phenotypic assortment in animal social networks: Weighted associations are more robust than binary edges. Animal Behaviour, 89, 141–153. 10.1016/j.anbehav.2014.01.001

[mee313741-bib-0023] Farine, D. R. (2017). A guide to null models for animal social network analysis. Methods in Ecology and Evolution, 8, 1309–1320. 10.1111/2041-210X.12772 29104749 PMC5656331

[mee313741-bib-0024] Farine, D. R. (2018). When to choose dynamic vs. static social network analysis. Journal of Animal Ecology, 87, 128–138. 10.1111/1365-2656.12764 28994101

[mee313741-bib-0025] Farine, D. R. , & Aplin, L. M. (2019). Spurious inference when comparing networks. Proceedings of the National Academy of Sciences of the United States of America, 116, 16674–16675. 10.1073/pnas.1900143116 31409703 PMC6708361

[mee313741-bib-0026] Farine, D. R. , Firth, J. A. , Aplin, L. M. , Crates, R. A. , Culina, A. , Garroway, C. J. , Hinde, C. A. , Kidd, L. R. , Milligan, N. D. , Psorakis, I. , Radersma, R. , Verhelst, B. , Voelkl, B. , & Sheldon, B. C. (2015). The role of social and ecological processes in structuring animal populations: A case study from automated tracking of wild birds. Royal Society Open Science, 2, 150057. 10.1098/rsos.150057 26064644 PMC4448873

[mee313741-bib-0027] Farine, D. R. , & Milburn, P. J. (2013). Social organisation of thornbill‐dominated mixed‐species flocks using social network analysis. Behavioral Ecology and Sociobiology, 67, 321–330. 10.1007/s00265-012-1452-y

[mee313741-bib-0028] Farine, D. R. , & Strandburg‐Peshkin, A. (2015). Estimating uncertainty and reliability of social network data using Bayesian inference. Royal Society Open Science, 2, 150367. 10.1098/rsos.150367 26473059 PMC4593693

[mee313741-bib-0029] Farine, D. R. , & Whitehead, H. (2015). Constructing, conducting, and interpreting animal social network analysis. Journal of Animal Ecology, 84, 1144–1163. 10.1111/1365-2656.12418 26172345 PMC4973823

[mee313741-bib-0031] Forstmeier, W. , & Schielzeth, H. (2011). Cryptic multiple hypotheses testing in linear models: Overestimated effect sizes and the winner's curse. Behavioral Ecology and Sociobiology, 65, 47–55. 10.1007/s00265-010-1038-5 21297852 PMC3015194

[mee313741-bib-0032] Forstmeier, W. , Wagenmakers, E. J. , & Parker, T. H. (2017). Detecting and avoiding likely false‐positive findings – A practical guide. Biological Reviews, 92, 1941–1968. 27879038 10.1111/brv.12315

[mee313741-bib-0033] Franks, D. W. , Weiss, M. N. , Silk, M. J. , Perryman, R. J. Y. , & Croft, D. P. (2021). Calculating effect sizes in animal social network analysis. Methods in Ecology and Evolution, 12, 33–41. 10.1111/2041-210X.13429 PMC9033095 35464674

[mee313741-bib-0034] Gimenez, O. , Mansilla, L. , Klaich, M. J. , Coscarella, M. A. , Pedraza, S. N. , & Crespo, E. A. (2019). Inferring animal social networks with imperfect detection. Ecological Modelling, 401, 69–74. 10.1016/j.ecolmodel.2019.04.001

[mee313741-bib-0035] Gotelli, N. J. , & Graves, G. R. (1996). Null models in ecology. Smithsonian Institution Press.

[mee313741-bib-0036] Hart, J. D. A. , Weiss, M. N. , Brent, L. J. N. , & Franks, D. W. (2021). Common permutation methods in animal social network analysis do not control for non‐independence. bioRxiv, 2021.2006.2004.447124. 10.1007/s00265-022-03254-x PMC9617964 36325506

[mee313741-bib-0037] Harvey, P. H. (1987). On the use of null hypotheses in biogeography. In M. H. Nitechi & A. Hoffman (Eds.), Neutral models in biology (pp. 109–118). Oxford University Press.

[mee313741-bib-0038] He, P. , Maldonado‐Chaparro, A. , & Farine, D. R. (2019). The role of habitat configuration in shaping social structure: A gap in studies of animal social complexity. Behavioral Ecology and Sociobiology, 73, 9. 10.1007/s00265-018-2602-7

[mee313741-bib-0039] Hobson, E. A. , Avery, M. L. , & Wright, T. F. (2013). An analytical framework for quantifying and testing patterns of temporal dynamics in social networks. Animal Behaviour, 85, 83–96. 10.1016/j.anbehav.2012.10.010

[mee313741-bib-0040] Hobson, E. A. , Silk, M. J. , Fefferman, N. H. , Larremore, D. B. , Rombach, P. , Shai, S. , & Pinter‐Wollman, N. (in press). A guide to choosing and implementing reference models for social network analysis. Biological Reviews. 10.1111/brv.12775 PMC9292850 34216192

[mee313741-bib-0041] Hoppitt, W. , & Farine, D. R. (2018). Association indices for quantifying social relationships: How to deal with missing observations of individuals or groups. Animal Behaviour, 136, 227–238. 10.1016/j.anbehav.2017.08.029

[mee313741-bib-0042] Ives, A. R. (2015). For testing the significance of regression coefficients, go ahead and log‐transform count data. Methods in Ecology and Evolution, 6, 828–835. 10.1111/2041-210X.12386

[mee313741-bib-0044] Langen, T. A. (1996). Social learning of a novel foraging skill by white‐throated magpie‐jays (*Calocitta formosa*, Corvidae): A field experiment. Ethology, 102, 157–166. 10.1111/j.1439-0310.1996.tb01113.x

[mee313741-bib-0045] Lusseau, D. , Whitehead, H. , & Gero, S. (2008). Incorporating uncertainty into the study of animal social networks. Animal Behaviour, 75, 1809–1815. 10.1016/j.anbehav.2007.10.029

[mee313741-bib-0046] Mantel, N. (1967). The detection of disease clustering and a generalized regression approach. Cancer Research, 27(2), 209–220. 6018555

[mee313741-bib-0047] Miller, E. T. , Farine, D. R. , & Trisos, C. H. (2017). Phylogenetic community structure metrics and null models: A review with new methods and software. Ecography, 40, 461–477. 10.1111/ecog.02070

[mee313741-bib-0048] Nakagawa, S. , & Cuthill, I. C. (2007). Effect size, confidence interval and statistical significance: A practical guide for biologists. Biological Reviews, 82, 591–605. 10.1111/j.1469-185X.2007.00027.x 17944619

[mee313741-bib-0049] O'Hara, R. B. , & Kotze, D. J. (2010). Do not log‐transform count data. Methods in Ecology and Evolution, 1, 118–122. 10.1111/j.2041-210X.2010.00021.x

[mee313741-bib-0050] Papageorgiou, D. , Christensen, C. , Gall, G. E. , Klarevas‐Irby, J. A. , Nyaguthii, B. , Couzin, I. D. , & Farine, D. R. (2019). The multilevel society of a small‐brained bird. Current Biology, 29, R1120–R1121. 10.1016/j.cub.2019.09.072 31689393

[mee313741-bib-0051] Platt, J. R. (1964). Strong inference. Science, 146, 347–353. 10.1126/science.146.3642.347 17739513

[mee313741-bib-0052] Puga‐Gonzalez, I. , Sueur, C. , & Sosa, S. (2021). Null models for animal social network analysis and data collected via focal sampling: Pre‐network or node network permutation? Methods in Ecology and Evolution, 12, 22–32. 10.1111/2041-210X.13400

[mee313741-bib-0053] Puth, M. T. , Neuhauser, M. , & Ruxton, G. D. (2015). On the variety of methods for calculating confidence intervals by bootstrapping. Journal of Animal Ecology, 84, 892–897. 10.1111/1365-2656.12382 26074184

[mee313741-bib-0054] Ripperger, S. P. , Carter, G. G. , Duda, N. , Koelpin, A. , Cassens, B. , Kapitza, R. , Josic, D. , Berrio‐Martinez, J. , Page, R. A. , & Mayer, F. (2019). Vampire bats that cooperate in the lab maintain their social networks in the wild. Current Biology, 29, 4139–4144. 10.1016/j.cub.2019.10.024 31679938

[mee313741-bib-0055] Ripperger, S. P. , Carter, G. G. , Page, R. A. , Duda, N. , Koelpin, A. , Weigel, R. , Hartmann, M. , Nowak, T. , Thielecke, J. , Schadhauser, M. , Robert, J. , Herbst, S. , Meyer‐Wegener, K. , Wagemann, P. , Schroder‐Preikschat, W. , Cassens, B. , Kapitza, R. , Dressler, F. , & Mayer, F. (2020). Thinking small: Next‐generation sensor networks close the size gap in vertebrate biologging. Plos Biology, 18, e3000655. 10.1371/journal.pbio.3000655 32240158 PMC7117662

[mee313741-bib-0056] Ryder, T. B. , Horton, B. M. , van den Tillaart, M. , Morales, J. D. , & Moore, I. T. (2012). Proximity data‐loggers increase the quantity and quality of social network data. Biology Letters, 8, 917–920. 10.1098/rsbl.2012.0536 22859558 PMC3497117

[mee313741-bib-0057] Sah, P. , Mendez, J. D. , & Bansal, S. (2019). A multi‐species repository of social networks. Scientific Data, 6, 44. 10.1038/s41597-019-0056-z 31036810 PMC6488576

[mee313741-bib-0058] Schielzeth, H. , & Forstmeier, W. (2009). Conclusions beyond support: Overconfident estimates in mixed models. Behavioral Ecology, 20, 416–420. 10.1093/beheco/arn145 19461866 PMC2657178

[mee313741-bib-0059] Spiegel, O. , Leu, S. T. , Sih, A. , & Bull, C. M. (2016). Socially‐interacting or indifferent neighbors? Randomization of movement paths to tease apart social preference and spatial constraints. Methods in Ecology and Evolution, 7, 971–979.

[mee313741-bib-0060] Sundaresan, S. R. , Fischhoff, I. R. , & Dushoff, J. (2009). Avoiding spurious findings of nonrandom social structure in association data. Animal Behaviour, 77, 1381–1385. 10.1016/j.anbehav.2009.01.021

[mee313741-bib-0061] Thomson, C. E. , Winney, I. S. , Salles, O. C. , & Pujol, B. (2018). A guide to using a multiple‐matrix animal model to disentangle genetic and nongenetic causes of phenotypic variance. PLoS ONE, 13, e0197720. 10.1371/journal.pone.0197720 30312317 PMC6193571

[mee313741-bib-0062] Webber, Q. M. R. , Schneider, D. C. , & Vander Wal, E. (2020). Is less more? A commentary on the practice of 'metric hacking' in animal social network analysis. Animal Behaviour, 168, 109–120. 10.1016/j.anbehav.2020.08.011

[mee313741-bib-0063] Webber, Q. M. R. , & Vander Wal, E. (2018). An evolutionary framework outlining the integration of individual social and spatial ecology. Journal of Animal Ecology, 87, 113–127. 10.1111/1365-2656.12773 29055050

[mee313741-bib-0064] Weiss, M. N. , Franks, D. W. , Brent, L. J. N. , Ellis, S. , Silk, M. J. , & Croft, D. P. (2021). Common datastream permutations of animal social network data are not appropriate for hypothesis testing using regression models. Methods in Ecology and Evolution, 12(2), 255–265. 10.1111/2041-210X.13508 35464674 PMC9033095

[mee313741-bib-0065] Whitehead, H. (2008). Analyzing animal societies. University of Chicago Press.

[mee313741-bib-0066] Whitehead, H. , Bejder, L. , & Ottensmeyer, C. A. (2005). Testing association patterns: Issues arising and extensions. Animal Behaviour, 69, e1–e6. 10.1016/j.anbehav.2004.11.004

[mee313741-bib-0067] Whitehead, H. , & James, R. (2015). Generalized affiliation indices extract affiliations from social network data. Methods in Ecology and Evolution, 6, 836–844. 10.1111/2041-210X.12383

