# Supplementary material for: Permutation tests for hypothesis testing with animal social network data: Problems and potential solutions
Source: Methods Ecol Evol. 2021 Oct 28;13(1):144–56. doi: 10.1111/2041-210X.13741 (PMC9297917; doi:10.1111/2041-210X.13741)
Supplement: Supplementary file 1 — Supplementary Material [file MEE3-13-144-s001.pdf]

SUPPLEMENTAL TEXT AND FIGURES FOR:

Permutation tests for hypothesis testing with animal social network data: problems and potential solutions

Damien R. Farine[1,2,3] and Gerald G. Carter[4,5]

[1]Department of Evolutionary Biology and Environmental Studies, University of Zurich, Zurich, Switzerland.
[2]Department of Collective Behavior, Max Planck Institute of Animal Behavior, Konstanz, Germany.
[3]Centre for the Advanced Study of Animal Behaviour, University of Konstanz, Germany.
[4]Department of Ecology, Evolution, and Organismal Biology, The Ohio State University, Columbus, USA
[5]Smithsonian Tropical Research Institute, Balboa, Ançon, Panama

Correspondence: damien.farine@ieu.uzh.ch , *Phone: +41 44 635 4767*

ILLUSTRATING THE DRIVERS OF TYPE I AND TYPE II ERRORS

Here we clarify the main problem by considering a simple, verbal, but concrete example of why errors can arise when using permutation tests. Imagine a study population where animals cluster for warmth each night. Clustering associations include 2-10 individuals and the group composition can change from night to night. The dataset contains a list of observed clusters, their location, time, and the individuals in those clusters that could be correctly identified. From these data, researchers generate a network describing the propensity for each dyad to be observed in the same cluster with the aim of finding out if kin are more likely to cluster: do individuals preferentially cluster with kin?

First, the researchers consider a node permutation approach (e.g. using a Mantel or MRQAP test) to correlate pairwise kinship and association. However, kin can associate more than expected by chance without kin-biased behaviors or even kin discrimination—for example if siblings are born at the same time, are limited to similar home ranges, and then disperse at around the same time. Under such a scenario, a significant result from a node permutation test would correctly support the hypothesis that the network is kin-structured, but it would be spurious support for the hypothesis that kinship is a causal driver of social associations (false positive). Alternatively, kinship-biased association could be undetected or underestimated (false negative) if younger animals are both more likely to associate with kin but also less likely to be individually marked and recorded. In the presence of sampling bias or other nuisance effects, hypotheses about the process generating an effect of kinship on association could be challenging to accurately assess using basic node permutation tests (but see Alternative Approaches section for more discussion on how node permutations can be restricted to help alleviate some of these effects).

The researchers might therefore turn to using a pre-network permutation test. To create expected outcomes for the relationship between kinship and edge weights, they repeatedly swap individual observations within sampled locations and time periods. Doing so allows them to eliminate the nonsocial nuisance effects described above, but it also assumes that no other social factors cause social network structure, because pre-network permutations assume a null hypothesis of no social effects at all. Imagine that in this species, individuals always spend about 90% of their time with only 1-3 closely bonded associates. This constraint on social structure means that every node (individual) always has a large variance in edge weights (association rates) across all its possible associates, because most association rates are zeros while a few are larger (i.e. closer to one). This variance in edge weight is much greater than expected from random associations (few zeros but also few larger values). When the researchers create randomised networks using pre-network permutations, many zero association rates are replaced with non-zero values as individuals that never co-occurred are swapped into groups together, and the individuals in the resulting random networks almost never spend 90% of their time with a few individuals (i.e. the previously strong edges become weaker). Even if association rates are not kin-biased and the close social bonds are randomly distributed with regards to kinship, the extremely nonrandom social structure of the observed network could easily lead to a false positive with regards to the effect of kinship (Weiss *et al.* 2021). If even a few strong bonds exist among kin by chance in the observed data, these strong kin bonds might not ever appear in the randomized networks. This scenario occurs because the observed network typically has much higher variance in the measure of interest (edge weight or node metric) than the corresponding random networks from pre-network permutations (Aplin *et al.* 2015; Firth *et al.* 2018; Weiss *et al.* 2021). Thus, once again, the actual effect of kinship on association is challenging to accurately evaluate in the presence of a nuisance effect (in this case social preferences unrelated to kinship).

A different potential problem with pre-network permutation tests is that they can provide overly-confident estimates of minor deviations from random. In part, this problem occurs because constructing social networks requires large numbers of observations (Langen 1996; Farine & Strandburg-Peshkin 2015; Davis, Crofoot & Farine 2018) with many repeated observations of the same individuals, and in any hypothesis test a sufficiently large number of observations can invariably produce p-values well below 0.05 even when the effect size is not biologically important.

Pre-network permutations can also suffer from incorrect inference when networks contain only a few nodes. For instance, consider a simplistic network containing only three nodes (male, male and female), and with only three observed dyadic associations, with all three being between the two males. In such a scenario, a pre-network permutation testing whether females are less connected than expected by chance would be significant at $p<0.05$, as the observed data is the only one of 27 combinations of the three dyadic associations that would result in the female having a degree of zero (the p-value here would approach 0.037). While any inference drawn from three observations of three individuals is obviously unreliable, the same problem can be present, and much harder to notice, in more complex analyses.

The previous example illustrates problems caused by nuisance effects when the variables are traits of edges (kinship and association). The same principles apply when testing hypotheses relating the traits of individuals. For instance, a false impression that individuals of one sex are more gregarious (higher network centrality) could be caused by sex-based differences in observability, attraction to spatially clumped resources, variation in home-range size, differential survival in different habitats, or differences in the timing of their presence or absence in the population. These issues are common to many other types of ecological studies, but their influence can be exacerbated in social network studies. Some of these effects are sampling effects, and others will only cause a problem if there's a mismatch between the question of interest to the researcher and the question being addressed by the analysis (e.g. 'do males prefer larger groups?' versus 'do males occur in larger groups?', see Figure S1).

Although it is useful to conceptually distinguish between effects that are social versus non-social, or biological versus methodological, these can be difficult to disentangle in practice, because biological biases can influence methodological biases and social behaviours can contribute to the apparently 'non-social' effects. Studies could benefit from making more explicit considerations of the social decisions that contribute to spatial and temporal biases. For example, individuals' spatial ranges could be determined by density-dependence in their decisions to settle in a location or move elsewhere. We provide some advice on how permutation tests can help uncover such effects in the future directions section.

Another example is how social behaviour can influence observability. Consider a study testing if male birds have higher degree centrality. If females tend to be found at the periphery of groups, then an observer standing close to the centre of a group might be more likely to miss observations of females and their associates, whereas the observer can always detect the associates of the (predominately male) individuals at the centre of a group, thereby introducing a sex bias in the number of observed associates. An observer standing outside the group could have the opposite bias. This simple example highlights how biases can arise even when observations are made in groups where every individual is individually-identifiable, and why the focus on detecting relationships (i.e. estimating edges) makes network studies more prone to nuisance effects than many other types of studies.

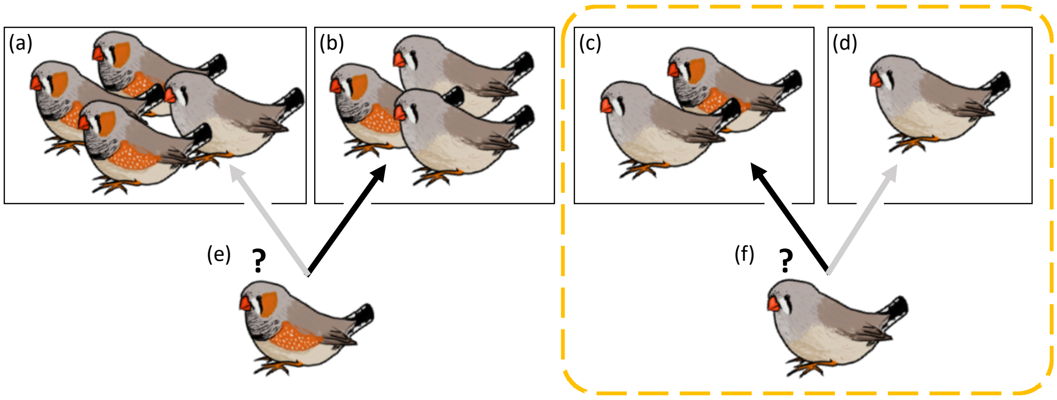

Figure S1. Example of the challenges associated with testing hypotheses on social data. Compared to females, are males more often in larger groups? In the scenario shown above, males (colourful individuals) are observed in larger groups (e.g. groups a, b, c) more often than are females (less colourful individuals). However, now consider a slightly different question: Compared to females, do males have a greater preference for larger groups? The same observation is not conclusive regarding the causal processes. Imagine that groups a and b are outside a territory (shown by the dashed line) defended by a single dominant male (shown in c) that allows females to enter freely and form groups (c and d) but excludes other males. Even if all males choose smaller groups (b vs a), and all females (e.g. f) choose larger groups (c vs d) given their available options, we could still observe males in larger groups than females due to the constraints imposed by the territory. Thus, even if males are equally or less gregarious, they can end up in larger groups and having more social connections. However, by permuting the individual observations within locations (a pre-network permutation), we can simulate individuals of both sexes making random social decisions given the options that were available to them. Under such a null model, a subordinate male that was never seen in the territory (groups c and d) would never be observed there in the permuted data, and would therefore always be found in larger groups. Because males would end up in larger groups in both the observed and permuted datasets, this pre-network permutation test would correctly indicate that males do not prefer larger groups. However, the same test would incorrectly fail to detect that males and females differ in their numbers of connections. The former question might be the focus of a study on how individual decisions shape network structure, whereas the latter question might be the focus of a study of how network structure shapes information or pathogen transmission.

An advantage of pre-network permutations is that they allow precise control over many possible nuisance effects, without needing to measure or even identify all of them. For example, if a lower proportion of individuals are individually-identifiable at the edge of a study area, then individuals seen at the edge would always occur in groups containing fewer individuals that can be individually-identified. In this case, controlling for space would automatically control for differences in group size that arise due to spatial variation in identification rates by only swapping individuals into other groups that also had fewer individual-identified individuals. Such an effect could be challenging to control for explicitly in a statistical model. On the other hand, pre-network permutation tests allow many other important aspects of the permuted networks to change (such as degree distributions or variances), and thus pre-network permutations alone should not be used to assess the effect of a predictor while controlling for social structure (Puga-Gonzalez, Sueur & Sosa 2021; Weiss *et al.* 2021). Ideally, one would use pre-network permutations to control for nuisance effects without making unrealistic random networks, which would include maintaining the variance in association strengths.

POTENTIAL IMPROVEMENTS OF THE DOUBLE PERMUTATION TEST

There are many further directions and refinements that can be explored for the double-permutation test. While we have demonstrated that the method performs adequately across a range of scenarios using simulated data, it is not always possible to simulate all possible types of uncertainty that might exist in empirical datasets. For example, the median expected value might not effectively represent the value expected by the null hypothesis, because when the possible configurations of the data are severely limited (for instance by a small sample size of observations per individual), the resulting distribution of expected metric values might not be unimodal. For example, if individuals have strong group size preferences, then their expected degree might jump dramatically from their preferred values to the distribution of mean degrees from the population, as more and more swaps are made. It can therefore be useful to visualize the expected distributions of metric values across individuals when possible, and to remove individuals that have been under-sampled (Farine & Strandburg-Peshkin 2015).

Rather than using a single value (such as the median, see Figure 2), future studies could explore ways of carrying over uncertainty from the distribution of permutation values when calculating the corrected test statistic. For example, one could use a Monte Carlo approach that repeatedly samples from the distribution of permuted values when calculating the deviation score for each unit in the analysis (each node or each edge), and use these many measurements to estimate the 95% range of the corrected test statistic. Carrying uncertainty through the analysis could be implemented using a Bayesian framework (Farine & Strandburg-Peshkin 2015), or to model the dynamic process of the observation of connections in the network (Koskinen & Snijders 2007). Thus, there remains significant grounds for continued improvements in the methods for hypothesis testing using animal social network data.

For many studies, it is important to not only test a hypothesis of interest but also to accurately estimate the connection strength between individuals. One method that has been proposed are generalized affiliation indices (Whitehead & James 2015). These involve regressing the observed association strength against nuisance factors (such as home-range overlap) to generate a corrected value that accounts for the opportunity to associate. Permutation tests have also been suggested as a means of identifying nonrandom preferred or avoided relationships (Whitehead, Bejder & Ottensmeyer 2005). Yet, it remains to be determined whether permutation tests could also provide more accurate estimates of the strength of each relationship. Following these methods, it could be possible to estimate a corrected association (or interaction) strength by subtracting some measure of the distribution of permuted values from the observed value of each edge.

SIMULATION 1 DETAILS

The first simulation starts by drawing N individual trait values T_i from a normal distribution with a mean of 0 and a standard deviation of 2. We assign each individual to have on average K observations by drawing K_i from a Poisson distribution with $\lambda = K$ and balancing these values to ensure that $\sum K_i = N \times K$. We then create G groups, where $G = 0.5 \times N \times K$, and randomly assign each of these groups to have a group size value X ranging from 1 to 10. To allocate individuals into groups, we order the individuals from the smallest trait value to the largest to create scenarios where the trait value should impact the social behaviour of individuals (trait has social impact, $T_S = TRUE$), or order these at random to create scenarios where the trait value has no relation to the social behaviour of individuals ($T_S = FALSE$). We assign each individual into groups by selecting the K_i groups that have empty spaces, and with a higher probability of selecting smaller groups. In doing so, individuals earlier in the order are disproportionately more likely to be assigned to smaller groups, filling them up, and leaving only larger groups for later individuals to fill, thereby creating a relationship between individuals' trait value T_i and their weighted degree D_i when T_S is true. Because the simulations did not always create a relationship (especially in simulations of eigenvector centrality and betweenness), we stochastically re-ordered the traits when $T_S = TRUE$ and no relationship was present to create a relationship. This was done by ordering individuals by their trait value and probabilistically allocating them to nodes according to the node values, thereby resulting in a strong relationship.

From these observation data, we follow the design of our double permutation method (see Figure 2) to first calculate how each node's degree deviates from what is expected from random behaviour, and, second, to calculate the relationship between the deviance score for weighted degree values D_i' and the trait values T_i. We then implement two conceptual variations, L_S, to go with the two T_S scenarios above. In the first variant (location effect, $L_S = TRUE$), we assume that X corresponds to a spatial preference, such that individuals prefer patches closest to the centre of their home ranges in a one-dimensional linear environment ranging in values from 1 to 10. Patches at one end of this environment, corresponding to a larger X, contain more resources and therefore can hold more individuals. The second variant (no location effect, $L_S = FALSE$) is a scenario in which the group size value X corresponds to the outcome of social decisions, such that individuals with a larger X have a preference for larger groups. These variants enable us to use the same code to produce a relationship between T_i and network degree D_i where in one scenario the decisions are social ($T_S = TRUE$ and $L_S = FALSE$) and in another scenario the relationship between T_i and D_i arises from decisions that are driven by a non-social covariate ($T_S = TRUE$ and $L_S = TRUE$). To control for the location X_j that each group was observed in when $L_S = TRUE$, we use within-location swaps in the pre-network permutation tests. We also calculate individual's most common location to include as a covariate in "controlled node permutations".

SIMULATION 3 DETAILS

In simulation 3, we first create a 'real' network comprised of N individuals with a network density D drawn from a uniform distribution (ranging from 0.05 to 0.6), and selecting $Z \times D$ edges (where Z is the maximum possible number of undirected edges) with probabilities 0.6, 0.3, and 0.1 for edge types 1 to 3, respectively (and all other edges set to 0).

Second, we make an average of K observations per individual. Specifically, we create $1.2 \times \max(K_i)$ sampling periods (Whitehead 2008), and randomly allocate individuals to being observed in K_i of these sampling periods. To create associations for each sampling period, we then select all pairs of individuals with an edge present in the real network and where both are present in that sampling period, and draw a 0 or 1 to signify whether they were observed together or not. Here we set the binomial probability of being observed together when they are both present to be 0.1 for edges of weak associates, 0.6 for edges of preferred associates, and 0.9 for edges of strongly-bonded associates, based on the real network. We select the number of individuals and observations (N and K) using the same parameter sets as in model 1.

Third, we create a kinship network, setting the kinship level of each individual based on their edge type in the real network. Specifically, we draw relative kinship values from a beta distribution with $\alpha = 1$ and $\beta = 2$ (i.e. left-skewed) for missing edges and edges of weak associates, $\alpha = 2$ and $\beta = 2$ (i.e. unskewed) for edges of preferred associates, and $\alpha = 3$ and $\beta = 2$ (i.e. right-skewed) for edges of strongly-bonded associates. Because beta distributions range from 0 to 1, these distributions assume that 1 corresponds to the closest relatives in the population. We use these parameters to create kinship distributions with differences in mean relative kinship (0.33, 0.5, 0.6, respectively) according to social relationship type. Note that these relative kinship values can be divided by two to create a maximum kinship value of 0.5 with no effect on the outputs of the model.

Our simulations comprise two scenarios. In the first scenario, edge weights are unrelated to kinship, which we achieve by randomizing the kinship matrix after generating it (thereby keeping the same relationship between the variance in the network edges and in the kinship matrix). In the second scenario, associations are kin-biased by keeping the kinship matrix as it was generated, thus strongly-bonded associates have the highest kinship on average and weak associates and non-associates (missing edges) have on average the lowest kinship. We create the networks, conduct the pre-network permutation tests, and conduct the regressions using the MRQAP functionality in the R package *asnipe* (Farine 2013).

SUPPLEMENTARY FIGURES

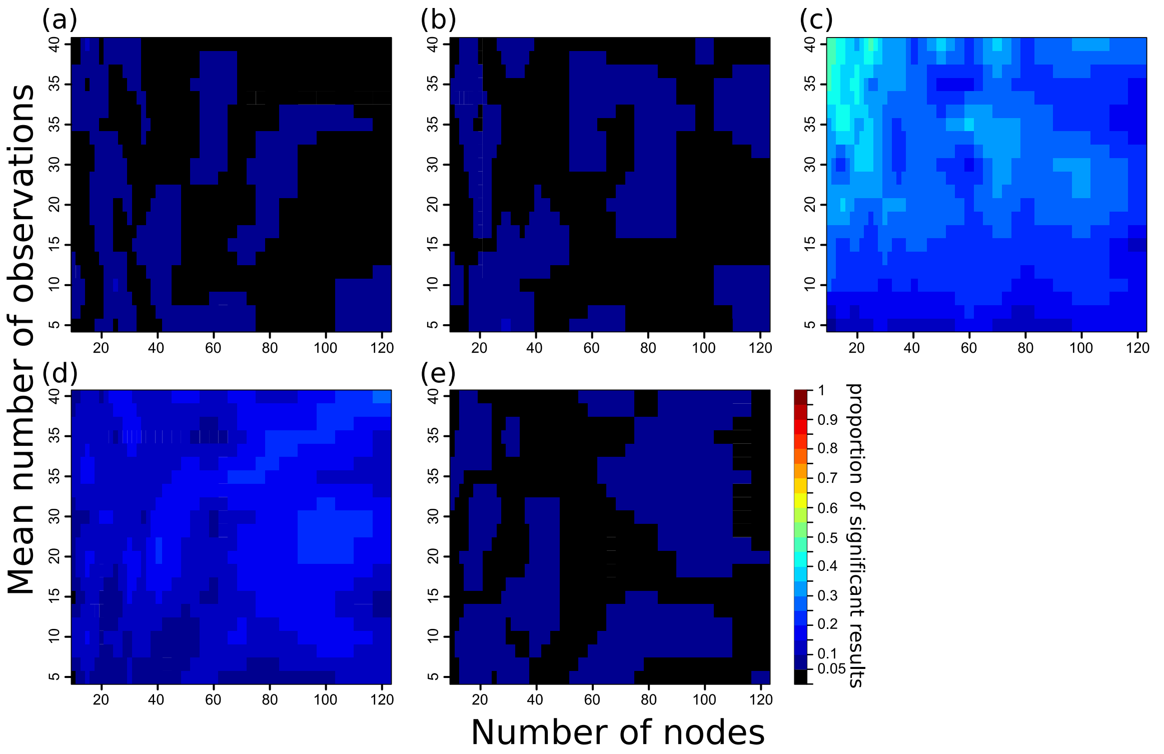

Figure S2. Proportions of statistically significant results for differently-sized networks and different observation efforts for simulation 1 under the scenario when $T_S = FALSE$ (no effect is present) and $L_S = FALSE$ (individuals are not preferentially located in different patches), with degree as the network metric. Panels represent the p-values calculated using (a) node permutation tests with the coefficient value as the test statistic, (b) node permutation tests controlling for number of observations, (c) pre-network permutation tests with the coefficient value as the test statistic, (d) pre-network permutation tests with the t statistic as the test statistic, and (e) double permutation tests with the coefficient as the test statistic. These results highlight the propensity for pre-network permutation tests (c) to produce spurious results when networks have few nodes but many observations (top left of the plot).

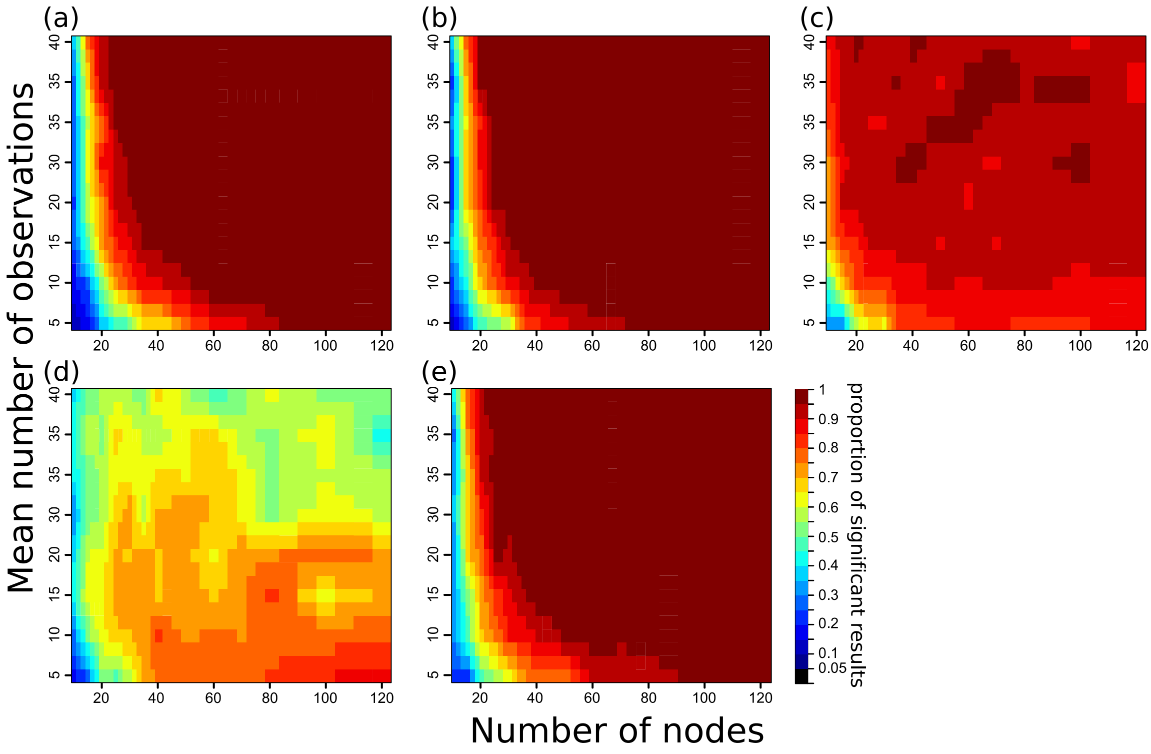

Figure S3. Proportions of statistically significant results for differently-sized networks and different observation efforts for simulation 1 under the scenario when $T_S = TRUE$ (an effect is present) and $L_S = FALSE$ (the effect is not a spatial confound), with degree as the network metric. Panels represent the p-values calculated using (a) node permutation tests with the coefficient value as the test statistic, (b) node permutation tests controlling for number of observations, (c) pre-network permutation tests with the coefficient value as the test statistic, (d) pre-network permutation tests with the t statistic as the test statistic, and (e) double permutation tests with the coefficient as the test statistic. These results highlight the propensity for pre-network permutation tests (c) to be more likely to produce significant results when networks have few nodes but many observations (left-hand of the plot relative to panels a, b and e). Further, results show that using the t statistic (d) produces unreliable results (i.e. the significance does not increase when more observations are made).

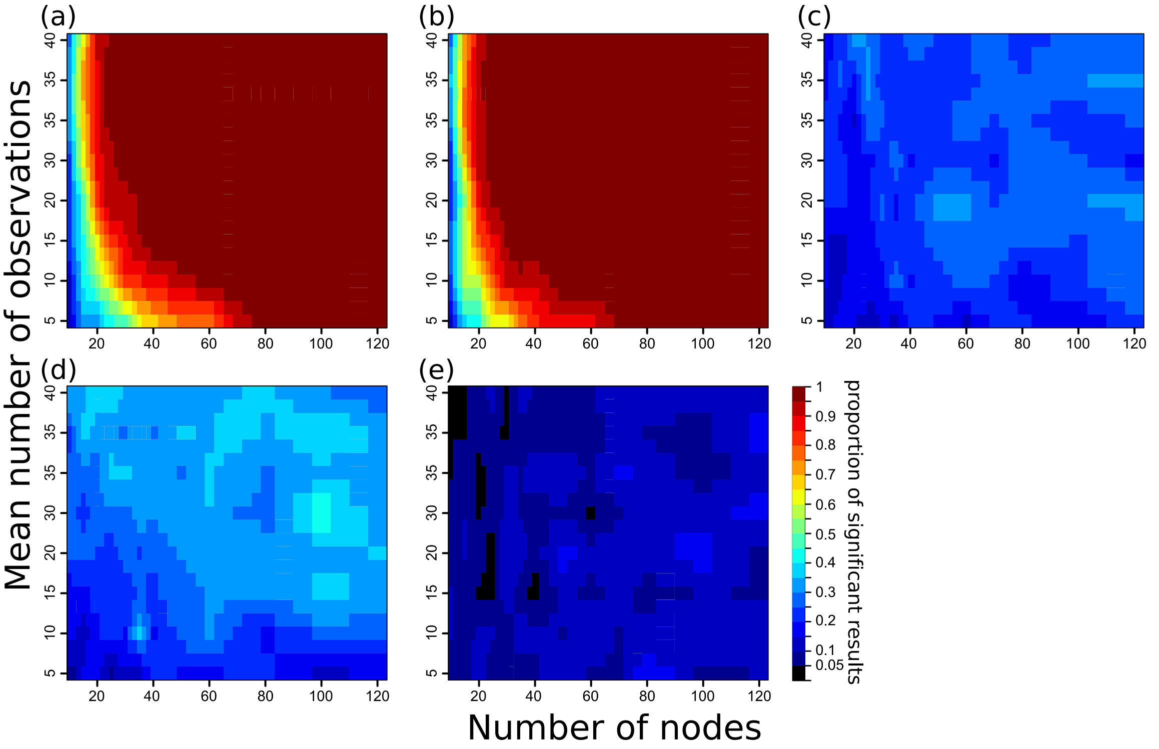

Figure S4. Proportions of statistically significant results for differently-sized networks and different observation efforts for simulation 1 under the scenario when $T_S = TRUE$ (an effect is present) and $L_S = TRUE$ (the effect is a spatial confound), with degree as the network metric. Panels represent the p-values calculated using (a) node permutation tests with the coefficient value as the test statistic, (b) node permutation tests controlling for number of observations, (c) pre-network permutation tests with the coefficient value as the test statistic, (d) pre-network permutation tests with the t statistic as the test statistic, and (e) double permutation tests with the coefficient as the test statistic. These results highlight the poor performance of node permutation-based models (panels a and b).

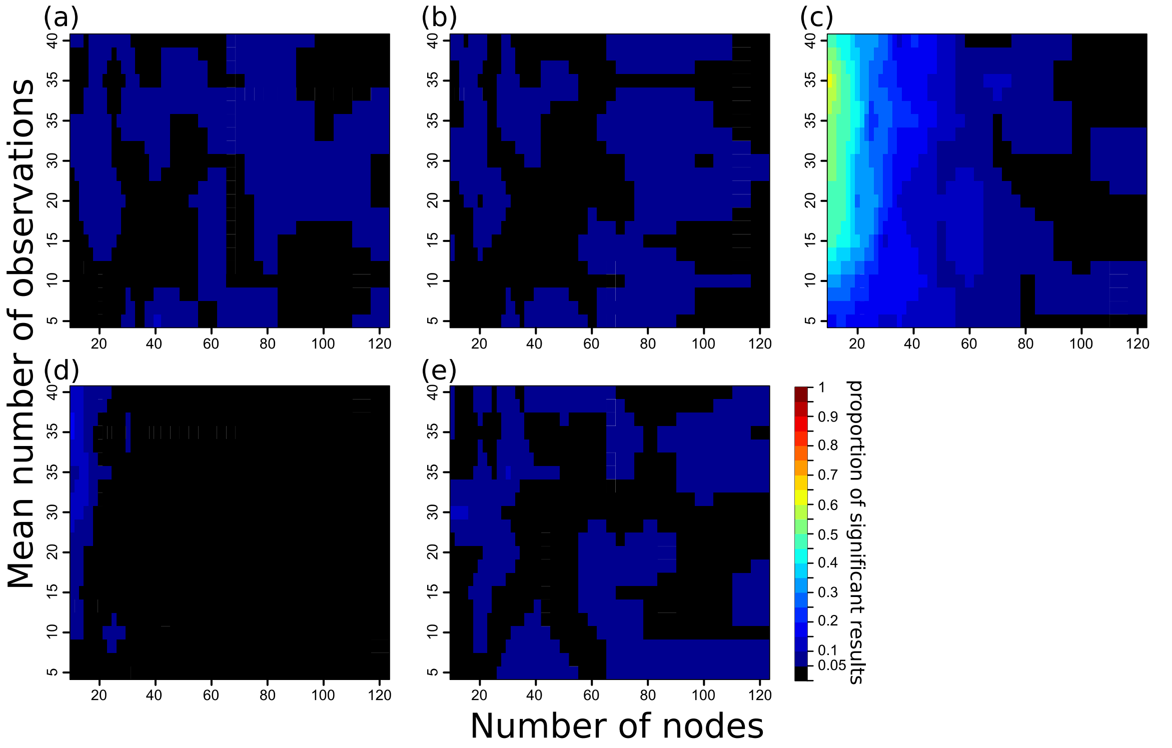

Figure S5. Proportions of statistically significant results for differently-sized networks and different observation efforts for simulation 3 under the scenario where kinship does not predict association rates (edge weights). Panels represent the p-values calculated using (a) node permutation tests with the coefficient value as the test statistic, (b) node permutation tests controlling for number of observations, (c) pre-network permutation tests with the coefficient value as the test statistic, (d) pre-network permutation tests with the t statistic as the test statistic, and (e) double permutation tests with the coefficient as the test statistic. These results highlight the propensity for pre-network permutation tests (c) to produce spurious results when networks have few nodes present (left-hand-side of the plot).

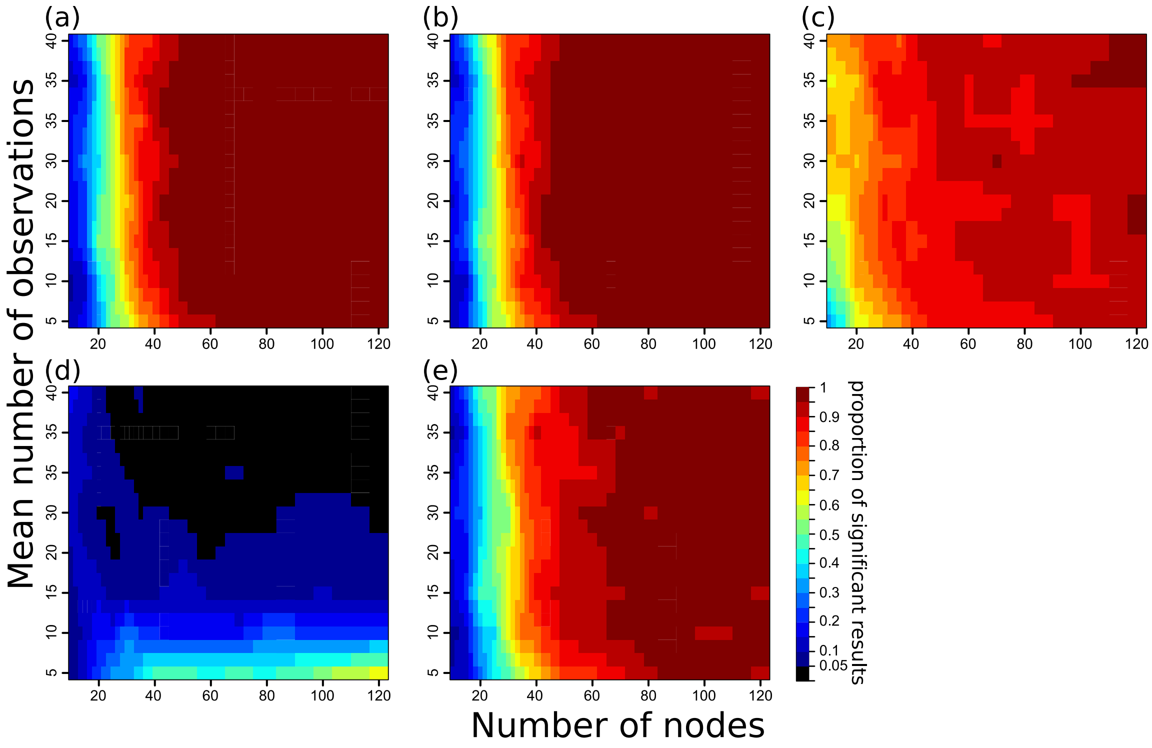

Figure S6. Proportions of statistically significant results for differently-sized networks and different observation efforts for simulation 3 under the scenario where kinship predicts association rates (edge weights). Panels represent the p-values calculated using (a) node permutation tests with the coefficient value as the test statistic, (b) node permutation tests controlling for number of observations, (c) pre-network permutation tests with the coefficient value as the test statistic, (d) pre-network permutation tests with the t statistic as the test statistic, and (e) double permutation tests with the coefficient as the test statistic. These results highlight the propensity for pre-network permutation tests (c) to be more likely to produce significant results when networks have few nodes but many observations (left-hand of the plot relative to panels a, b and e). Further, results show that using the t statistic (d) produces unreliable results (i.e. the significance does not increase with when more observations are made).

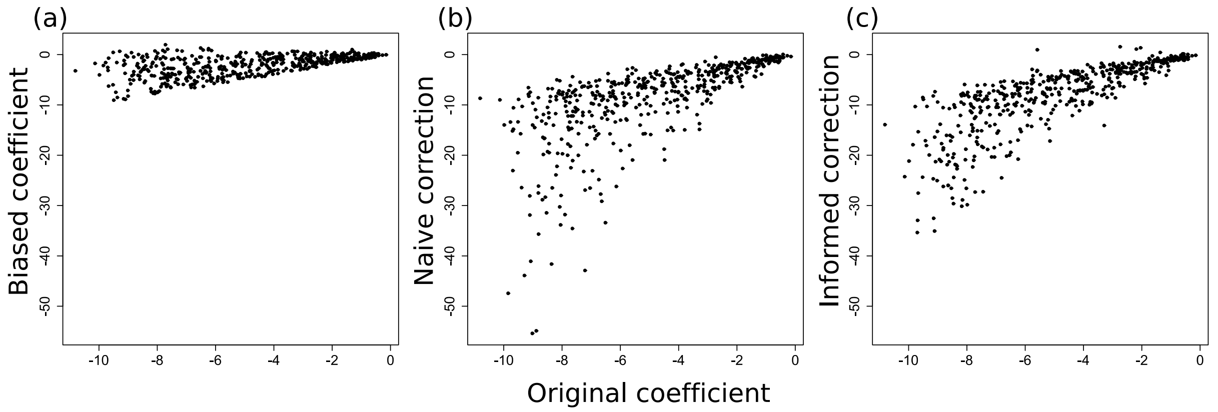

Figure S7. Relationship between the original coefficient value (the relationship between degree and sex prior to introducing an observation bias) and estimations of the coefficient value using the data from simulation 2 where an effect is present (females are more gregarious). (a) The original coefficient versus the coefficient estimated from the biased observations. (b) The original coefficient versus a naïve correction involving adding only the number of observation for each individual as a covariate in the model. (c) The original coefficient versus an informed correction that involves including an interaction term between sex and the number of observations. Each point represents one simulation. While these coefficients are correlated, the corrected coefficient values can be greatly over-estimated, suggesting that adding the number of observations into a model does not produce reliable effect sizes.

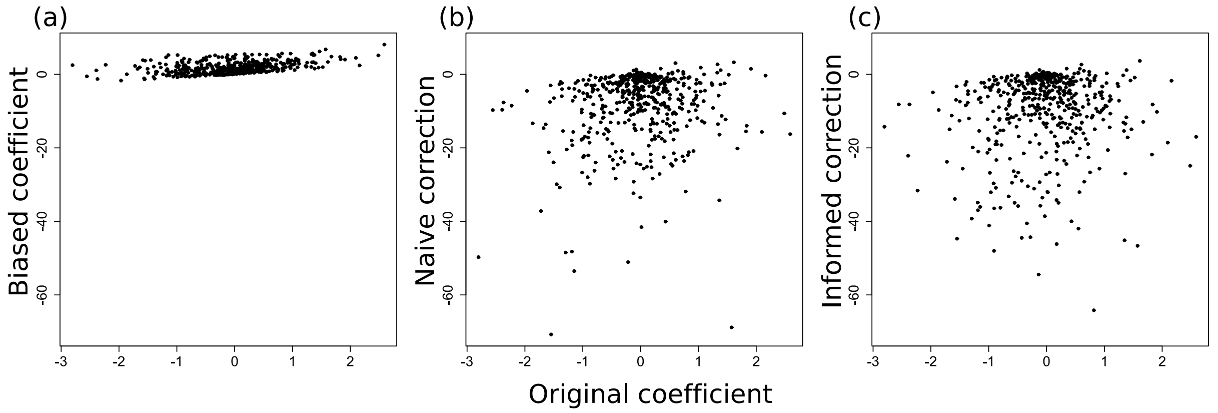

Figure S8. Relationship between the original coefficient value (the relationship between degree and sex prior to introducing an observation bias) and estimations of the coefficient value using the data from simulation 2 where no effect is present (females and males are equally gregarious). (a) The original coefficient versus the coefficient estimated from the biased observations. (b) The original coefficient versus a naïve correction involving adding only the number of observation for each individual as a covariate in the model. (c) The original coefficient versus an informed correction that involves including an interaction term between sex and the number of observations. Each point represents one simulation. Because there was no original effect present, the coefficients are not correlated. However, the corrected coefficient values generate extremely large coefficient values, suggesting that adding the number of observations into a model does not produce reliable effect sizes.

SUPPLEMENTAL REFERENCES

Aplin, L.M., Firth, J.A., Farine, D.R., Voelkl, B., Crates, R.A., Culina, A., Garroway, C.J., Hinde, C.A., Kidd, L.R., Psorakis, I., Milligan, N.D., Radersma, R., Verhelst, B. & Sheldon, B.C. (2015) Consistent individual differences in the social phenotypes of wild great tits (*Parus major*). *Animal Behaviour,* **108,** 117-127.

Davis, G.H., Crofoot, M.C. & Farine, D.R. (2018) Estimating the robustness and uncertainty of animal social networks using different observer methods. *Animal Behaviour,* **141,** 29-44.

Farine, D.R. (2013) Animal Social Network Inference and Permutations for Ecologists in R using asnipe. *Methods in Ecology and Evolution,* **4,** 1187–1194.

Farine, D.R. & Strandburg-Peshkin, A. (2015) Estimating uncertainty and reliability of social network data using Bayesian inference. *Royal Society Open Science,* **2,** 150367.

Firth, J.A., Cole, E.F., Ioannou, C.C., Quinn, J.L., Aplin, L.M., Culina, A., McMahon, K. & Sheldon, B.C. (2018) Personality shapes pair bonding in a wild bird social system. *Nature Ecology & Evolution,* **2,** 1696-1699.

Koskinen, J.H. & Snijders, T.A.B. (2007) Bayesian inference for dynamic social network data. *Journal of Statistical Planning and Inference,* **137,** 3930-3938.

Langen, T.A. (1996) Social learning of a novel foraging skill by white-throated magpie-jays (Calocitta formosa, Corvidae): A field experiment. *Ethology,* **102,** 157-166.

Puga-Gonzalez, I., Sueur, C. & Sosa, S. (2021) Null models for animal social network analysis and data collected via focal sampling: Pre-network or node network permutation? *Methods in Ecology and Evolution,* **12,** 22-32.

Weiss, M.N., Franks, D.W., Brent, L.J.N., Ellis, S., Silk, M.J. & Croft, D.P. (2021) Common datastream permutations of animal social network data are not appropriate for hypothesis testing using regression models. *Methods in Ecology and Evolution,* **10.1111/2041-210X.13508**.

Whitehead, H. (2008) *Analyzing animal societies*. University of Chicago Press, Chicago, USA.

Whitehead, H., Bejder, L. & Ottensmeyer, C.A. (2005) Testing association patterns: issues arising and extensions. *Animal Behaviour,* **69,** e1-e6.

Whitehead, H. & James, R. (2015) Generalized affiliation indices extract affiliations from social network data. *Methods in Ecology and Evolution,* **6,** 836-844.
